# YKT6 expression, exosome release, and survival in non-small cell lung cancer

**DOI:** 10.18632/oncotarget.9862

**Published:** 2016-06-06

**Authors:** Marc Ruiz-Martinez, Alfons Navarro, Ramón M. Marrades, Nuria Viñolas, Sandra Santasusagna, Carmen Muñoz, Josep Ramírez, Laureano Molins, Mariano Monzo

**Affiliations:** ^1^ Molecular Oncology and Embryology Laboratory, Department of Human Anatomy and Embryology, School of Medicine, University of Barcelona, IDIBAPS, Barcelona, Spain; ^2^ Department of Pneumology, Institut Clínic del Tórax (ICT), Hospital Clinic de Barcelona, University of Barcelona, IDIBAPS, CIBER de Enfermedades Respiratorias (CIBERES), Barcelona, Spain; ^3^ Department of Medical Oncology, Institut Clinic Malalties Hemato-Oncològiques (ICMHO), Hospital Clinic de Barcelona, University of Barcelona, IDIBAPS, Barcelona, Spain; ^4^ Department of Pathology, Centro de Diagnóstico Biomédico (CDB), Hospital Clinic de Barcelona, University of Barcelona, IDIBAPS, CIBERES, Barcelona, Spain; ^5^ Department of Thoracic Surgery, Institut Clínic del Tórax (ICT), Hospital Clinic de Barcelona, University of Barcelona, Barcelona, Spain

**Keywords:** YKT6, exosomes, NSCLC, miR-134, miR-135b

## Abstract

**Background:**

Cancer-derived exosomes are involved in metastasis. YKT6 is a SNARE protein that participates in the regulation of exosome production and release, but its role in non-small cell lung cancer (NSCLC) has not been examined.

**Materials and Methods:**

Ultracentrifugation-purified exosomes from the A549 cell line were studied by CRYO-TEM, nanoparticle tracking analysis and western blot (TSG101 marker). YKT6 was inhibited using a DsiRNA and selected pre-microRNAs. MicroRNAs targeting YKT6 were validated by Renilla/Luciferase assay and western blot. YKT6 expression and its prognostic impact were analyzed in 98 tissue specimens from resected NSCLC patients.

**Results:**

Membranous nanosized vesicles (mode size: 128nm) with TSG101 protein were purified from A549 cells. YKT6 inhibition reduced exosome release by 80.9%. We validated miR-134 and miR-135b as miRNAs targeting YKT6, and transfection with the pre-miRNAs also produced a significant reduction in exosome release. The analysis of YKT6 in tumor samples showed that patients with high levels had shorter disease-free and overall survival.

**Conclusions:**

YKT6 is a key molecule in the regulation of exosome release in lung cancer cells and is in turn precisely regulated by miR-134 and miR-135b. Moreover, YKT6 levels impact prognosis of resected NSCLC patients.

## INTRODUCTION

Exosomes are small vesicles (40-100nm) released by cells are crucial to normal and pathological intercellular communication [1]. They can contain functional proteins and nucleic acids, including non-coding RNAs [2]. In cancer cells, exosomes can take part in different functions, including proximal and distal regulation [3]. Cancer cells are able to modulate the microenvironment through the release of exosomes, which participate in the modification of the surrounding stroma [4]. Moreover, cancer-derived exosomes can protect tumor cells by inhibiting the recognition of cancer cells by the immune system [5]. Interestingly, through exosomes, cancer cells can transfer mutant genes such as K-RAS, which can promote malignization of the recipient cells [6]. In this line, evidence suggests that tumor-derived exosomes can participate in the formation of the premetastatic niche [7].

Exosome biogenesis starts with the formation of intraluminal vesicles in endosomal compartments. This results in a multivesicular body that needs to be transported and fused with the plasma membrane to release the exosomes [1, 8]. Exosome production and release is precisely regulated by several proteins, including Rab [9–11] and SNARE (Soluble N-ethylmaleimide-Sensitive-Factor Attachment Receptor) family proteins [4]. Increasing evidence suggests that tumor cells release an excessive amount of exosomes, which may influence tumor initiation, growth, progression, metastasis, and drug resistance [3]. EPI64, which specifically activates Rab27a, has been related to the regulation of exosome release in the lung cancer cell line A549 [12]. The study of proteins involved in exosome production and secretion is a promising source of biomarkers in cancer.

YKT6 is a SNARE protein involved in the mechanisms of cell membrane fusion, associated with vesicular transit [13], and it has been identified as a key protein for release of WNT3A-containing exosomes in HEK293 cells [14]. In breast cancer cells, YKT6 overexpression was associated with an aggressive phenotype *in vitro,* and with the ability of breast epithelia to metastasize when injected intravenously into mice [15]. Interestingly, in breast cancer patient samples, YKT6 was upregulated in p53-mutated tumors that were resistant to docetaxel, while the *in vitro* silencing of YKT6 in breast cancer cells enhanced docetaxel-induced apoptosis [16]. However, the possible role of YKT6 as a prognostic marker has not been examined in other tumors, including non-small-cell lung cancer (NSCLC), where prognosis is dismal, with 5-year survival rates of 19-50% in surgically resected patients [17].

MicroRNAs (miRNAs) are small non-coding RNAs which negatively regulate translation by binding to their 3′UTR mRNA target [18]. miRNAs are involved in the regulation of different biological processes, such as cell proliferation, differentiation and apoptosis [18]. Although several miRNAs have been predicted to target YKT6, to the best of our knowledge, none has yet been validated.

In order to further clarify the role of YKT6 and its regulating miRNAs in the release of exosomes in NSCLC, we have studied YKT6 inhibition *in vitro* and examined its effect on exosome release. In addition, we have examined the impact of YKT6 expression in tumor samples on outcome of resected NSCLC patients.

## RESULTS

### Exosome purification and YKT6 inhibition in the A549 cell line

**Figure 1:**
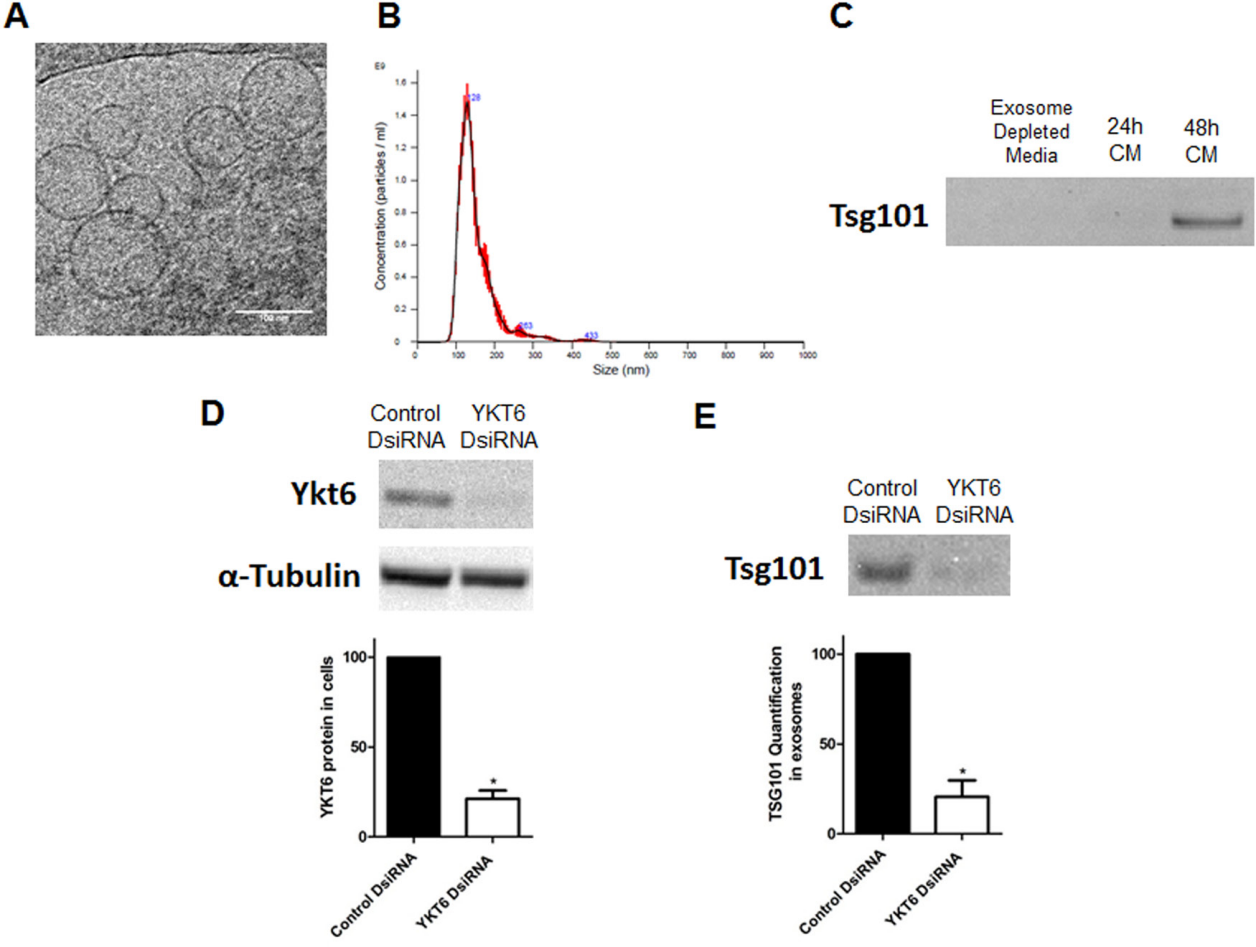
Exosome characterization, and YKT6 inhibition and effect on exosome release **A.** Electronic microscopic image of exosomes identified by Cryo-TEM; **B.** Results of nanoparticle tracking analysis of exosomes; **C.** Western Blot using TSG101 marker in exosome depleted media used as negative control and 24h and 48h supernatant from A549 cell line cultured with exosome depleted media; **D.** Western blots of A549 cells transfected with control or YKT6 DsiRNA and quantification to relative loading control α-tubulin; **E.** Western blot of exosomes isolated from A549 cells transfected with control or YKT6 DsiRNA and quantification of the exosomal marker TSG101. All experiments were performed in triplicate and data was shown as mean ± SEM. * p<0.05.

To verify that exosomes were correctly purified, we studied the exosome product obtained from supernatant of the A549 cell line by three methods: cryo-TEM, nanoparticle tracking analysis (NTA), and western blot using the exosomal marker TSG101. Cryo-TEM identified membranous nano-sized vesicles (Figure 1A). NTA showed a uniform population of nanoparticles from exosome isolations with a mode of 128nm (130.6 +/− 3.5nm) (Figure 1B). Finally, western blot showed clear expression of TSG101 in our samples (Figure 1C).

Both the mRNA and the protein of YKT6 were detected in the A549 cell line and YKT6 inhibition using a DsiRNA resulted in a 78.8% reduction of YKT6 protein levels in comparison with control cells (p=0.023) (Figure 1D). We then studied whether YKT6 inhibition decreases exosome release. Interestingly, cells with YKT6 inhibited released 80.9% fewer exosomes than control cells (p=0.013), as measured by western blot with the exosomal marker TSG101 (Figure 1E).

### miRNA regulation of YKT6 and exosome release

**Figure 2:**
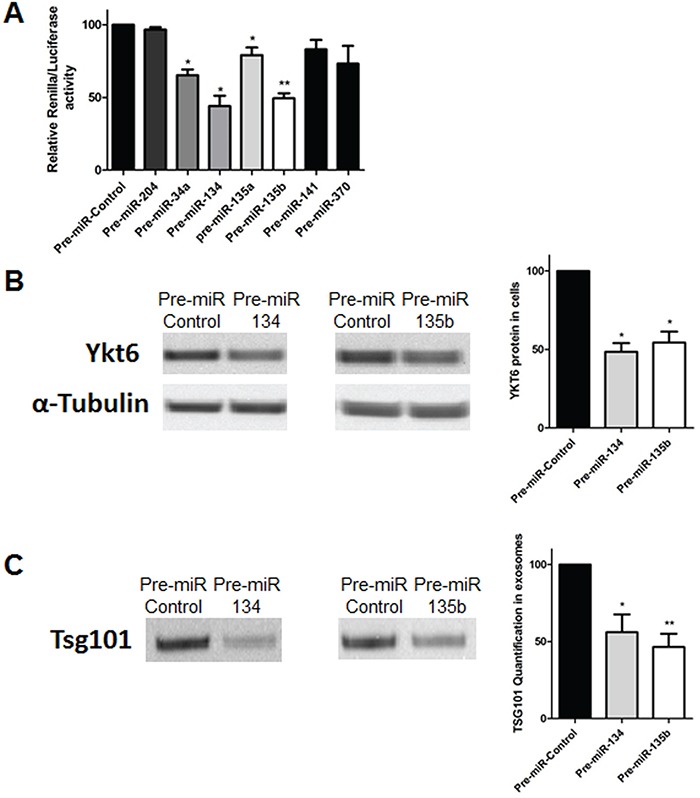
YKT6 inhibition by miRNAs and effect on exosome release **A.** Renilla luciferase activity in A549 cells transfected with selected miRNAs and control. **B.** Western blots of A549 cells transfected with control, pre-miR-134 or pre-miR-135b and quantification of YKT6 signal relative to loading control α-tubulin. **C.** Western blot of exosomes isolated from A549 cells transfected with control, pre-miR-134 or pre-miR-135b and quantification of the exosomal marker TSG101. All experiments were performed in triplicate and data was shown as mean ± SEM. * p<0.05; **p<0.01.

Using TargetScan and miRò databases, six miRNAs were selected as potential miRNAs targeting YKT6: miR-34a, miR-141, miR-134, miR-135a, miR-135b and miR-370. To validate that these miRNAs target YKT6, we performed a *Renilla/Luciferase* assay. The *Renilla/Luciferase* assay showed no significant differences between cells transfected with pre-miR-204, which is not predicted to target YKT6, and pre-miRNA negative control. However, in comparison with control, *Renilla luciferase* activity was 34.75%, 56.01%, 20.85% and 50.61% lower with pre-miR-34a (p=0.019), pre-miR-134 (p=0.022), pre-miR-135a (p=0.02) and pre-miR-135b (p=0.002), respectively. No significant differences were detected in cells transfected with pre-miR-370 or pre-miR-141 (Figure 2A).

Since the greatest decrease in *Renilla luciferase* activity was associated with miR-134 and miR-135b, these two miRNAs were selected for further study. We evaluated by western blot if changes in miR-134 and miR-135b levels correlated with changes in YKT6 protein levels. After transfection with pre-miRNAs, a reduction of 51.45% and 45.53% of YKT6 protein levels was observed for miR-134 (p=0.011) and miR-135b (p=0.022) (Figure 2B).

Finally, we evaluated whether these miRNAs could impact exosome release. Western blot analysis showed that in comparison with control, exosome release decreased by 43.92% in cells transfected with pre-miR-134 (p=0.032) and by 53.43% in cells transfected with miR-135b (p=0.008) (Figure 2C).

### YKT6, miR-134 and miR-135b expression in patient samples

All 98 patients included in the study had pathologically confirmed stage I-III NSCLC. The majority were males, and 87% had Eastern Cooperative Oncology Group (ECOG) performance status (PS) 1. Only 33.7% received adjuvant therapy. The main characteristics of the patients are described in Table 1.

**Table 1:** Patient characteristics and univariate p-value for disease-free survival (DFS) and overall survival (OS) in 98 patients with non-small-cell lung cancer

Characteristics	Value	N (%)	DFS	OS
**Sex**	Male	77 (78.6)	0.0741	0.0267
	Female	21 (21.4)		
**Age, yrs**	Mean (Range)	68 (33 - 83)	0.198	0.251
	<=65	43 (43.9)		
	>65	55 (56.1)		
**ECOG PS**	0	9 (9.2)	0.6906	0.3695
	1	89 (88.8)		
**Stage**	I	58 (59.2)	0.0096	0.0866
	II	21 (21.4)		
	III	19 (19.4)		
**Histology**	Adenocarcinoma	51 (52)	0.2393	0.2714
	Squamous cell carcinoma	39 (39.8)		
	Others	8 (8.2)		
**Type of surgery**	Lobectomy/Bilobectomy	84 (85.7)	0.873	0.5719
	Pneumonectomy	7 (7.1)		
	Atypical resection	7 (7.1)		
**Smoking history**	Current smoker	33 (33.7)	0.6209	0.0819
	Former Smoker	52 (53.1)		
	Never smoker	10 (10.2)		
	Unknown	3 (3.1)		
**Adjuvant treatment**	Yes	33 (33.7)	0.6137	0.6564
	No	65 (66.3)		
**Relapse**	No	62 (63.3)		
	Yes	36 (36.7)		
***TP53* mutations**	Yes	22 (22.4)	0.6207	0.9094
	No	73 (74.5)		
	Unknown	3 (3.1)		
***KRAS* mutations**	Yes	17 (17.3)	0.1826	0.1056
	No	79 (80.6)		
	Unknown	2 (2)		

ECOG, Eastern Cooperative Oncology Group; PS, performance status

**Figure 3:**
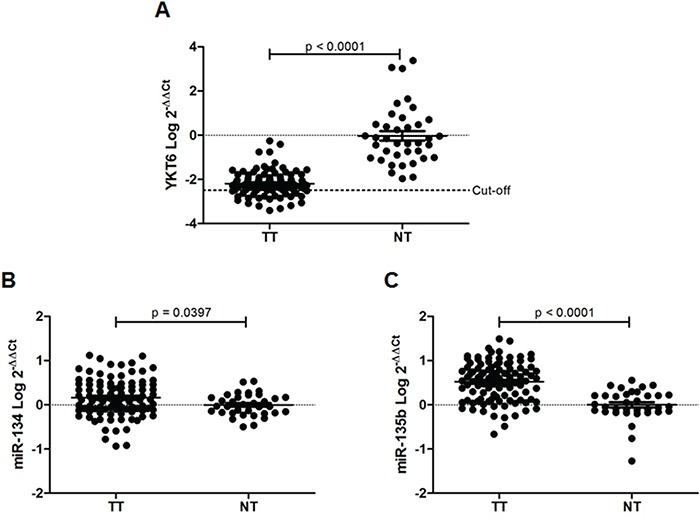
YKT6, miR-134 and miR-135b in tissue samples from NSCLC patients **A.** YKT6, **B.** miR-134, and **C.** miR-135 expression in 98 tumor tissue (TT) and 38 normal tissue (NT) samples from patients with NSCLC.

RealTime-PCR analysis showed that YKT6 was expressed at lower levels in tumor than in normal tissue (p<0.0001) (Figure 3A). In contrast, both miR-134 (p<0.0397) and miR-135b (p<0.001) were expressed at higher levels in tumor than in normal tissue (Figure 3B-3C). When we performed a correlation analysis between miR-134 and miR-135b expression and YKT6 expression, we could not identify a significant correlation between YKT6 and the two miRNAs in tumor tissue. However, in the paired normal tissue, we observed a significant inverse correlation between miR-135b and YKT6 (r=−0.368, p=0.045).

### YKT6 mRNA expression, patient outcome, and exosome release

**Figure 4:**
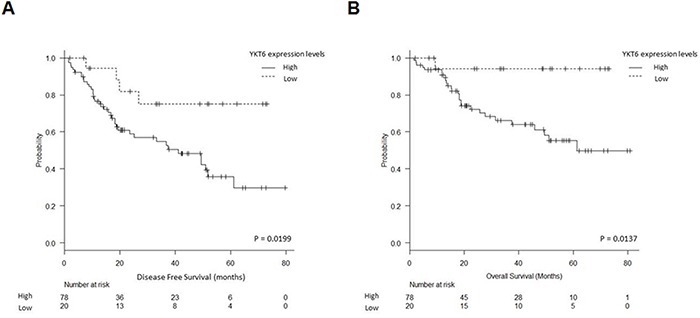
YKT6 expression and patient outcome **A.** Disease-free survival and **B.** overall survival in 98 NSCLC patients according to YKT6 mRNA expression levels.

Only YKT6 expression was associated with clinical outcome. According to the optimal cutoff determined by MaxStat (80th percentile), patients were classified in two groups: high (n=78) and low (n=20) YKT6 levels. Mean disease-free survival (DFS) was 42.1 months (95%CI, 34.5-49.7) for patients with high YKT6 expression and 59.5 months (95%CI, 47.9-71.1) for those with low expression (p=0.0199) (Figure 4A). Mean overall survival (OS) was 54.07 months (95%CI, 46.3-61.8) for patients with high YKT6 expression, compared to 69.3 months (95% CI, 62.2-76.4) for those with low expression (p=0.0137) (Figure 4B). No association was found between YKT6 expression levels in tumor and any clinicopathological or molecular characteristics.

**Figure 5:**
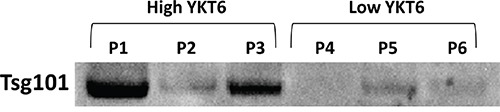
Exosome quantification in plasma from NSCLC patients Six patients from the studied cohort with available plasma samples at diagnosis were selected for exosome analysis. Three of them were classified as high and three as low according to YKT6 levels in their tumor tissue.

Since high levels of YKT6 may lead to increased release of exosomes and hence poorer prognosis, in an exploratory analysis, we studied the plasma exosome levels of six patients of our cohort with available samples. The patients were classified according to their tumor YKT6 levels as high (n=3) or low (n=3). Patients with high YKT6 levels had more exosomes in plasma than those with low levels (Figure 5).

## DISCUSSION

In the present study, we have studied exosome production and release in the A549 cell line and observed how the inhibition of the SNARE protein YKT6 produced a crucial downregulation in overall exosome levels. Moreover, we have examined the miRNA-mediated regulation of YKT6 and identified miRNAs able to inhibit YKT6 translation and thus indirectly modulate exosome release. Finally, we studied YKT6 expression in NSCLC patient samples and found that patients with high levels of YKT6 had shorter DFS and OS.

Cancer-derived exosomes are promising markers for diagnosis and prognosis in cancer patients. Exosome release has been shown to be a potential prognostic marker in some tumors, such as colorectal cancer, where the level of circulating exosomes correlates with poor prognosis and shorter survival [19]. In lung cancer, exosomal EGFR protein levels have been postulated as a biomarker for lung cancer diagnosis [20]. Proteins involved in the regulation of exosome production and release in tumor cells, such as SNARE proteins, could be surrogate markers of exosome levels. SNARE proteins are key mediators in membrane fusion events in the secretory pathway [21, 22]. YKT6, a unique SNARE protein that is highly conserved from yeast to human [23], has been observed in cytosol, membrane and perinuclear locations in cells and has been implicated in multiple steps of vesicle transport in yeast. Taken together, these findings suggest that YKT6 is a tightly regulated key protein in exosome release [23, 24]. Here, we have shown that inhibition of YKT6 greatly affects exosome release in the lung cancer cell line A549. Moreover, we have identified two miRNAs that participate in the regulation of YKT6 levels: miR-134 and miR-135b. Furthermore, inhibition of YKT6 resulting from increasing miR-134 and miR-135b levels decreases overall exosome levels in the cells.

In NSCLC patient samples, YKT6 mRNA levels were downregulated in tumor compared to normal lung tissue, while miR-134 and miR-135b levels were upregulated in tumor compared to normal tissue, suggesting that the differences in YKT6 expression could be due to the differential expression of the regulatory miRNAs. However, an alternative explanation could be related to the hypoxic microenvironment produced by rapid tumor growth. Under hypoxic conditions, several genes, including YKT6, suffer changes in their expression levels. For example, YKT6 expression is significantly downregulated in hypoxic conditions (1% O_2_) in human renal proximal tubule epithelial cells [25].

To the best of our knowledge, no previous study has compared YKT6 expression in tumor and paired normal tissue, but the RAB family protein Rab27B, which is also involved in the regulation of exosome production and release, was downregulated in tumor samples from hepatocellular carcinoma [26]. In line with our results, miR-135b was upregulated in highly invasive NSCLC cells [27] and miR-134 was upregulated in lung tumors, though no correlation was found between miR-134 and clinicopathological characteristics or survival [28].

Few studies have examined the impact of YKT6 expression on outcome in cancer patients. Our finding that high YKT6 expression was associated with poor prognosis is in line with previous studies in breast cancer, where YKT6 was identified as a gene that may be linked to invasive phenotypes and to tumorigenesis in breast cancer cell lines [15]. Moreover, high YKT6 expression has also been postulated as a mechanism of docetaxel resistance in p53-mutated breast tumors [16]. High levels of YKT6 in the tumor may lead to increased release of exosomes, leading to a more aggressive phenotype and poorer prognosis, since it is known that cancer-derived exosomes are involved in the invasive phenotype and in the formation of the premetastatic niche [7]. Among a small subset of our patients, those with high YKT6 levels had more exosomes in plasma than those with low levels. Although these preliminary results require further validation with a greater number of plasma samples, they provide the first indications that YKT6 levels in the tumor may act as a surrogate of exosome levels in plasma. Of note, other genes related to exosome release have also been previously related to cancer; for example, high Rab27B levels have been linked to poor prognosis in bladder [29], pancreatic [30] and colorectal cancer [31] and hepatocellular carcinoma [26].

In conclusion, the present study is a first step towards the identification of coding genes and miRNAs involved in the regulation of exosome release that can influence prognosis in NSCLC. We have shown that YKT6 downregulation is associated with a remarkable reduction in exosome release in an NSCLC cell line and that low YKT6 expression is associated with better clinical outcome in NSCLC patients. Moreover, we have identified several miRNAs that regulate YKT6 levels. The role of these miRNAs and YKT6 in exosome release and patient outcome warrants further investigation.

## MATERIALS AND METHODS

### Exosome purification

A549 cells were cultured in DMEM (Gibco) supplemented with + 10% exosome-depleted FBS (Life Technologies) and 5% penicillin-streptomycin. Exosome isolation was performed from 48h conditioned media (6ml) by sequential centrifugation method at 4°C (300G 5′, 2,500G 20′, 10,000G 30′) followed by Ultracentrifugation 100,000G 2h, pellet washed with DPBS and ultracentrifuged again 100,000G 1h (Optima L-100 XP Ultracentrifuge with 70.1Ti Rotor and Policarbonate Tubes, Beackman Coulter).

### Exosome characteritzation and quantification

PBS resuspended exosomes were studied by cryo transmission electron microscopy (cryo-TEM) in a Jeol JEM 2011 transmission electron microscope at the Microscope Facility of the Autonomous University of Barcelona. To study the size of the purified vesicles nanoparticle tracking analysis (NTA) on a NanoSight LM10 (Malvern Instruments Ltd, Malvern, UK) was used and analyzed with NTA 3.0 software (Malvern).

To quantify the overall exosome product, samples were resuspended in LDS Sample buffer with reducing agent (Invitrogen) and analyzed by Western blot using the exosome marker TSG101 [32]. The whole product of purified exosomes were separated by sodium dodecyl sulfate–polyacrylamide electrophoresis in Bolt 4-12% BisTris gels under reducing conditions and transferred to PVDF membranes with I-Blot (Life Technologies). Membranes were incubated with mouse monoclonal TSG101 antibody (ab83, Abcam). Antibody binding was revealed by incubation with antimouse IgG HRP conjugate secondary antibody (A 9044, Sigma-Aldrich). Chemiluminescence was detected using Novex ECL Chemiluminescent Substrate Reagent Kit and read in Chemidoc System (Bio-Rad). The protein density of the bands was quantified using Quantity One software.

### YKT6 inhibition

50nM of control DsiRNA (Negative Control DS NC1, Integrated DNA Technologies[IDT]) or YKT6 DsiRNA (HSC.RNAI.N006555, IDT) were transfected on the A549 cells using lipofectamine 2000 (Invitrogen) as per manufacturer's protocol. 48h after transfection cells were lysated for protein extraction and YKT6 protein levels were quantified by Western blot.

### Identification and validation of miRNAs targeting YKT6

TargetScan (www.targetscan.org) and miRò (http://ferrolab.dmi.unict.it/index.html) databases were used to identify putative miRNAs targeting YKT6. To validate the potential miRNAs identified a *Renilla/Luciferase* assay was performed in A549 cells using a modified psiCHECK-2 vector with the 3′UTR region of YKT6. The 3′UTR region of *YKT6* was amplified using primers with Sgf and PmeI restriction sites (underniled): Forward primer 5′-GCGATCGCCGGAAACAAAACTCATGCT-3′; Reverse Primer 5′-GTTTAAACCCCTGAAGCACAAAGAAAGC-3′. The PCR product was cloned into a TOPO TA vector using TOP10F′ *Escherichia coli* competent cells (Invitrogen). Positive clones were selected by Kanamycin resistance and verified by Sanger sequencing. The selected clones were then digested and cloned into psiCHECK-2 vector. The modified psiCHECK-2 vector was confirmed by sequencing.

To perform the *Renilla/Luciferase* assay cells were transfected with 500ng of the modified psiCHECK-2 vector and 100nM of selected pre-miRNA/pre-miR miRNA Negative Control #2 (Invitrogen). *Renilla luciferase* and *Firefly luciferase* activity was measured 24h after transfection with the Promega Dual Luciferase Reporter Assay System (Promega) in a luminometer Versa Max Microplate Reader.

The effect of the miRNAs shown to be significant in the Renilla/Luciferase assay were then confirmed by Western blot and selected for further study.

### Western blot

Western Blot analysis was performed as previously described [33] using the following primary antibodies: YKT6 (H00010652-M03, Tebu-bio) and α-tubulin (T 6074, Sigma-Aldrich).

### Patient tissue samples

Between 2007 and 2013, tumor tissue samples were prospectively collected from 98 NSCLC patients who underwent complete surgical resection in our institution. In addition, paired normal tissue samples from 38 of these patients were also available. Samples were collected immediately after surgery, frozen at −80°C, and kept for further processing. Approval for the study was obtained from the institutional ethics committee of the Hospital Clinic of Barcelona, Spain. Written informed consent was obtained from each participant in accordance with the Declaration of Helsinki.

### RNA extraction and mRNA and miRNA quantification

Total RNA was extracted using Trizol total RNA isolation reagent (Invitrogen) as per the manufacturer's protocol. Total RNA was reverse transcribed with High-Capacity cDNA Reverse Transcription Kit (Applied Biosystems). cDNA obtained was used for quantitative real-time PCR in Step One Time PCR System for YKT6 (Hs01127135_m1) (Applied Biosystems). Cycle thresholds (Ct) for YKT6 were normalized to Ct for RNA18S (Hs99999901_s1), and relative quantification was calculated using 2^−ΔΔCt^.

miR-134 and miR-135b expression was assessed using TaqMan MicroRNA Assays (miR-134: 00459; 135b: 000461) (Life Technologies) as per the manufacturer's instructions. miRNA Ct were normalized with miR-191 Ct.

### Statistical methods

Comparison of continuous values was performed with Student's t-test. DFS was calculated from the time of surgical treatment to the date of relapse or death from any cause and OS was calculated from the time of surgical treatment to the date of death from any cause. DFS and OS were calculated using the Kaplan-Meier method. Optimal cut-off points of YKT6 mRNA and miRNAs expression were assessed by means of maximally selected log-rank statistics using the Maxstat package (version 2.8.1; R statistical package). All analyses were performed R version 2.13. Statistical significance was set at p≤0.05.
